# Um Olhar sobre o *Stress* nas Mulheres com Infarto Agudo do Miocárdio

**DOI:** 10.36660/abc.20190282

**Published:** 2020-10-13

**Authors:** Karine Schmidt, Aline da Silva Lima, Kelly Rocha Schmitt, Maria Antonieta Moraes, Marcia Moura Schmidt

**Affiliations:** 1 Instituto de Cardiologia Porto AlegreRS Brasil Instituto de Cardiologia, Porto Alegre, RS – Brasil

**Keywords:** Mulheres, Infarto do Miocárdio, Estresse Psicológico, Doença da Artéria Coronariana, Doenças Vasculares, Fatores de Risco

## Abstract

**Fundamento:**

As mulheres parecem ser mais suscetíveis ao estresse psicossocial quando comparadas aos homens, e o estresse está associado a piora na evolução clínica dos pacientes após o infarto agudo do miocárdio (IAM).

**Objetivos:**

Investigar se o sexo feminino é preditor independente de risco para o estresse e comparar os níveis de estresse entre mulheres e homens com IAM.

**Métodos:**

Estudo transversal de uma série de casos, realizado entre janeiro de 2017 e junho de 2018. Pacientes com idade entre 18 e 65 anos, atendidos na instituição por IAM nesse período. A existência de estresse foi avaliada por meio do Inventário de Sintomas de Stress para Adultos de LIPP (ISSL), que o categoriza em quatro fases: alerta, resistência, quase exaustão e exaustão, com base em uma lista de sintomas físicos e psicológicos. Os dados foram analisados pelo programa estatístico *Statistical Package for Social Sciences* (SPSS) versão 24.0. O nível de significância adotado foi um valor de p < 0,05.

**Resultados:**

Dos 330 entrevistados, 89% das mulheres e 70% dos homens apresentaram estresse; o sexo feminino quase triplicou as chances de sofrê-lo (EXP (B) 2,79; p = 0,02). Quanto às quatro fases, as mulheres mostraram-se mais em quase exaustão e exaustão, e os homens, mais em resistência.

**Conclusões:**

Este estudo evidenciou que as mulheres se encontram na terceira e quarta fases do estresse, ou seja, em situações de estresse psicossocial duradouras. Tais resultados podem auxiliar no desenvolvimento de estratégias específicas para prevenção e promoção da saúde conforme os sexos, visando minimizar os efeitos do estresse nesses pacientes.

## Introdução

*Selye* foi o primeiro a identificar o estresse como um conjunto de reações que o organismo apresenta frente a situações nas quais é exigido um esforço para a adaptação a elas.^1^ O estresse é entendido, então, como uma reação a qualquer evento estressor e pode desencadear sintomas comportamentais, psicológicos e físicos.^2^

No estudo intercontinental Interheart,^3^ realizado com 11.119 casos e 13.648 controles em 52 países, foi demonstrado que a presença de estressores dobra o risco de IAM. Os dados de um Inquérito Nacional de Saúde americano^4^ confirmam esses achados e demonstram ainda que o estresse ou a angústia psicológica podem duplicar o risco de IAM (RC = 2,0 [IC 95% 1,4 a 3,0]).

O estresse psicológico crônico provoca uma excessiva ativação do sistema nervoso simpático, levando à exacerbação da aterosclerose coronária e da disfunção endotelial.^5^^,^^6^ A longo prazo, pode também aumentar o risco de eventos coronários e morte.^7^

Estudos apontam que as mulheres são mais suscetíveis ao estresse psicossocial.^8^^,^^9^ Isso porque elas têm assumido uma vida cotidiana com múltiplos papéis sociais e familiares, tornando-se mais propensas a enfermidades como cardiopatias e doenças vasculares (tanto quanto os homens).^10^ O IAM e o acidente vascular cerebral (AVC) são as principais causadoras de morte em mulheres com mais de 50 anos, com mais óbitos por causas cardiovasculares do que outras, incluindo o câncer de mama.^11^

Em estudos brasileiros, as mulheres mais jovens apresentam maior frequência de sintomas de estresse quando comparadas aos homens.^12^^,^^13^ Contudo, nos estudos sobre estresse e doença isquêmica, o sexo feminino apresenta menor prevalência e casos em maior idade do que o sexo masculino.^14^^,^^15^ Tendo em vista o exposto, o objetivo deste estudo foi investigar os preditores de estresse, principalmente se o sexo feminino é preditor independente de risco, bem como comparar características sociodemográficas e clínicas, história pregressa e evolução intra-hospitalar, além dos níveis de estresse entre mulheres e homens com IAM.

## Método

### Delineamento e Participantes

Trata-se de um estudo transversal a partir de uma série de casos ao longo de 18 meses, cujos pacientes foram incluídos conforme os seguintes critérios: idade entre 18 e 65 anos, ou seja, idade ativa laboral; e atendidos por IAM com supradesnivelamento do segmento ST, menos de 12 horas de evolução, em um hospital de referência em cardiologia. Segundo a V Diretriz da Sociedade Brasileira de Cardiologia sobre o tratamento do IAM com supradesnivelamento do segmento ST (2015),^16^ o IAM é uma síndrome isquêmica aguda com supradesnivelamento do segmento ST > 1,0 mm em derivações contíguas no eletrocardiograma (ECG), e aumento e/ou queda dos níveis de marcadores cardíacos, principalmente da troponina (com valor acima do percentil 99), são essenciais para o diagnóstico. Os critérios de exclusão foram: delta T prolongado, necessidade de ventilação mecânica, ocorrência de *delirium* ou história prévia de demência, dificuldades cognitivas, ou ainda diagnóstico de doenças psiquiátricas, conforme o médico assistente, que impediram o entendimento e a assinatura do termo de consentimento.

Os participantes foram entrevistados nas primeiras 48 horas de internação, fornecendo dados sociodemográficos, história clínica pregressa e informações sobre fatores de risco para cardiopatia isquêmica. A classificação da raça baseou-se na autodeclaração deles. Hipertensão foi definida por diagnóstico prévio ou uso de anti-hipertensivos; dislipidemia foi considerada presente naqueles com diagnóstico anterior ou uso de hipolipemiantes; diabetes Melito foi definido pelo uso prévio de insulina ou substâncias hipoglicemiantes, ou glicemia de jejum documentada > 126 mg/dl em duas ocasiões. História familiar de doença arterial coronariana (DAC) foi considerada presente se parentes de primeiro grau tinham IAM ou morte por causa cardiovascular súbita antes dos 55 anos para os homens e 65 para as mulheres. Para o índice de massa corporal (IMC), foram levados em consideração peso e altura autorreferidos. Depressão foi estabelecida pela ocorrência de pelo menos um episódio maior de sintomas depressivos requerendo tratamento farmacológico; angina foi definida como dor ou desconforto em região anterior do tórax, epigástrio, mandíbula, ombro, dorso ou membros superiores, desencadeada ou agravada por atividade física ou estresse emocional, conforme classe II em diante, segundo as Diretrizes de Doença Coronária estável.^17^ Os prontuários médicos foram consultados para verificar a ocorrência de eventos na internação.

#### Instrumentos

A presença do estresse foi avaliada por meio do ISSL,^2^ instrumento validado pelo Conselho Federal de Psicologia. Ele é composto por uma lista de 53 sintomas físicos e psicológicos, divididos em períodos: nas últimas 24 horas, na última semana e no último mês. Além de informar a existência ou não de estresse, o instrumento categoriza-o em quatro fases: alerta (corresponde à pontuação obtida nos sintomas das últimas 24 horas); resistência e quase exaustão (pontuações da última semana) e exaustão (sintomas que estiveram presentes no último mês). O ISSL possibilita, assim, o diagnóstico do estresse, a verificação da fase em que a pessoa se encontra e a predominância de sintomas físicos, psicológicos ou mistos.

#### Fases do Estresse

A fase de alerta caracteriza-se por reações do sistema nervoso simpático logo que o organismo percebe o estressor. A de resistência apresenta-se quando um estressor permanece presente com o passar do tempo, fazendo com que a pessoa resista e busque forças para continuar lidando com o estresse, embora ainda tenha os sintomas. Na fase de quase exaustão, o processo de adoecimento se inicia, e os órgãos com maior vulnerabilidade genética ou adquirida passam a mostrar sinais de deterioração. Se não há alívio para o estresse com a remoção dos estressores ou o uso de estratégias de enfrentamento, ele chega à sua fase de exaustão, causando diversos problemas, como úlceras, gengivites, psoríase, hipertensão arterial, depressão, ansiedade, entre outros.^2^

#### Considerações Éticas

Esse estudo foi aprovado pelo Comitê de Ética Institucional (CAAE: 62727416.5.0000.5333). Todos os participantes aceitaram participar e assinaram o Termo de Consentimento Livre-Esclarecido (TCLE), conforme preceitos das Resoluções n. 466/12 e 510/2016 do CNS/MS.

#### Tamanho do Estudo

O cálculo do tamanho da amostra foi realizado com o Programa Win Pepi versão 11.29. Considerando-se que a diferença entre a proporção de homens e mulheres com estresse varia entre 30 e 50%^12^^,^^13^ e que 70% dos pacientes com IAM são do sexo masculino,^18^ com nível de significância de 0,05 e poder de 80, seriam necessários 194 participantes, sendo 114 homens e 80 mulheres.

#### Análise Estatística

Os dados foram registrados em um banco de dados no Excel e analisados pelo programa estatístico SPSS versão 24.0. O Teste Kolmogorov-Smirnov foi utilizado para verificação da normalidade das variáveis. As contínuas foram apresentadas por meio de média e desvio padrão, e as categóricas, por frequência absoluta e percentual. Foi realizada regressão logística multivariada para preditores de estresse com as variáveis que obtiveram p < 0,10 na análise bivariada. Para a comparação das variáveis entre os participantes com e sem estresse e conforme o sexo, utilizou-se o teste *t* para amostras independentes ou o teste qui-quadrado. Foi considerado um valor de p < 0,05 como estatisticamente significativo. A prevalência de estresse, as fases e a sintomatologia foram apresentadas em percentuais e comparadas com o qui-quadrado quando necessário, seguindo as normas do Manual do Instrumento, cujas respostas são categorizadas.^2^

## Resultados

**Figura 1:**
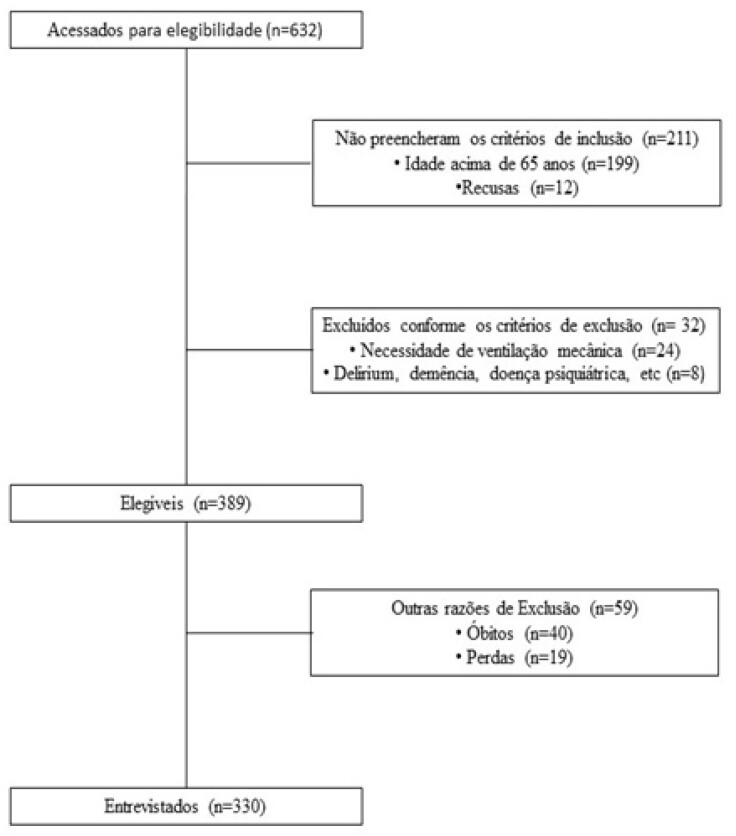
Fluxograma do estudo realizado no período de janeiro de 2017 a junho de 2018.

Os pacientes foram incluídos consecutivamente no período entre janeiro de 2017 e junho de 2018. Conforme o fluxograma do estudo, apresentado na Figura 1, dos 632 pacientes com IAM acessados para elegibilidade, 211 não preencheram os critérios de inclusão, 32 foram excluídos conforme os critérios estabelecidos, 40 foram a óbito e 19 foram excluídos por não terem sido entrevistados nas primeiras 48 horas da internação. A amostra, então, foi totalizada com 330 participantes.

Dos 330 entrevistados, 80 eram mulheres e 250 eram homens; 74% apresentaram estresse, sendo 89% das mulheres e 70% dos homens. Na Tabela 1 estão as características clínicas conforme a presença ou ausência de estresse. Foi observado que aqueles com estresse são, na maioria, mulheres, indivíduos com menos anos de estudo e internados pelo Sistema Único de Saúde (SUS). Não houve diferenças significativas entre os fatores de risco; entretanto, na história pregressa, os pacientes com estresse apresentaram mais angina e depressão. Também não houve diferenças quantos às intercorrências na internação desses pacientes.

**Tabela 1:** Diferença entre os gêneros na prevalência de estresse

Características	Total n = 330	Com estresse n = 245	Sem estresse n = 85	p*
**Características sociodemográficas**				
Sexo feminino (n, %)	80 (24,2%)	71 (29,0%)	9 (10,6%)	0,001
Idade (anos)	54,6 ± 7,7	54,4 ± 8,10	55,4 ± 7,0	0,28
IMC (kg/m²)	27,8 ± 5,1	27,6 ± 5,0	28,2 ± 5,5	0,33
Escolaridade (anos)	9,0 ± 4,0	8,7 ± 4,4	9,9 ± 3,3	0,02
Caucasianos (n, %)	249 (81,0%)	186 (81,0%)	63 (80,8%)	0,32
Renda familiar < 5 salários (n, %)	243 (76,0%)	186 (78,2%)	57 (69,5%)	0,11
SUS (n, %)	231 (76,0%)	184 (79,7%)	47 (64,4%)	0,008
**Fatores de risco**				
HAS (n, %)	180 (54,5%)	132 (54,0%)	48 (56,2%)	0,68
DM (n, %)	75 (22,7%)	55 (22,4%)	20 (23,5%)	0,84
TABAG (n, %)	120 (49,0%)	120 (49,0%)	38 (44,7%)	0,09
DSLP (n, %)	68 (27,8%)	68 (27,8%)	21 (24,7%)	0,58
HF + (n, %)	81 (24,8%)	62 (25,6%)	19 (22,4%)	0,55
**História clínica pregressa**				
IAM prévio (n, %)	64 (19,7%)	52 (21,7%)	12 (14,1%)	0,13
ICP prévia (n, %)	48 (14,8%)	40 (16,7%)	8 (9,4%)	0,10
AVC prévio (n, %)	20 (6,2%)	14 (5,8%)	6 7,1%)	0,68
ICC (n, %)	16 (5,0%)	12 (5,1%)	4 (4,7%)	0,90
Angina (n, %)	87 (26,8%)	74 (30,8%)	13 (15,3%)	0,005
DPOC (n, %)	9 (2,8%)	9 (3,8%)	0	–
IRC (n, %)	4 (1,2%)	3 (1,3%)	1 (1,2%)	0,95
Depressão (n, %)	51 (15,8%)	45 (19,0%)	6 (7,1%)	0,01
**Eventos intra-hospitalares**				
Arritmia (n, %)	9 (2,8%)	7 (3,0%)	2 (2,4%)	0,75
IAM recorrente (n, %)	2 (0,6%)	2 (0,9%)	0	–
AVC (n, %)	1 (0,3%)	1 (2,5%)	0	–
Óbito (n, %)	4 (1,3%)	4 (1,7%)	0	–

* Qui-quadrado ou t de student para amostras independentes.

IMC: índice de massa corporal; SUS: Sistema Único de Saúde; HAS: hipertensão arterial sistêmica; DM: diabete melito; TABAG: tabagismo; DSLP: dislipidemia; HF+: história familiar positiva para doença arterial coronariana; IAM: infarto agudo do miocárdio; ICP: intervenção coronariana percutânea; AVC: acidente vascular cerebral; ICC: insuficiência cardíaca congestiva; DPOC: doença pulmonar obstrutiva crônica; IRC: insuficiência renal crônica.

A partir da análise bivariada entre as características dos pacientes com e sem estresse, foram selecionadas aquelas com p < 0,10 para inclusão na análise multivariada (Tabela 2). Os resultados demonstraram que o sexo feminino e o tipo de assistência pública (SUS) foram os preditores independentes de estresse. O risco de exposição (Exp B) do sexo feminino foi de 2,79 (1,21 – 6,40, p = 0,02), indicando que ser mulher quase triplica a chance de sofrer estresse.

**Tabela 2:** Análise multivariada de preditores independentes de estresse

Características	Exp (B)	Intervalo de confiança (IC) 95%	p
Sexo feminino	2,79	1,21 a 6,40	0,02
Escolaridade	0,98	0,91 a 1,06	0,66
Angina	1,90	0,94 a 3,87	0,07
Depressão	1,52	0,57 a 4,02	0,40
Tabagismo	1,06	0,60 a 1,86	0,84
SUS	1,93	1,00 a 3,70	0,05

SUS: Sistema Único de Saúde.

A análise conforme o sexo incluiu somente os pacientes com estresse, totalizando 245 participantes (71 mulheres e 174 homens). Conforme observado na Tabela 3, as mulheres com estresse apresentam menor escolaridade do que os homens, e um percentual maior delas tem menor renda familiar. Elas também foram internadas pelo SUS com mais frequência que os homens (89,7% *versus* 75,5%; p = 0,014). Não foram encontradas diferenças significativas em relação à idade e ao IMC. Quanto aos fatores de risco, as mulheres apresentaram mais tabagismo do que os homens. Hipertensão, diabete melito e história familiar de doença coronariana foram semelhantes entre ambos os grupos. Parece haver uma tendência das mulheres à dislipidemia, e elas apresentaram mais história prévia de depressão e doença pulmonar obstrutiva crônica (DPOC) do que os homens. Na evolução intra-hospitalar, as mulheres apresentaram maior mortalidade.

**Tabela 3:** Comparação das características entre mulheres e homens com estresse

Características	Mulheres n = 71 (89%)	Homens n = 174 (70%)	p*
**Características sociodemográficas**			
Idade (anos)	55 ± 9	54 ± 7	0,667
IMC (kg/m²)	27 ± 7	28 ± 4	0,168
Escolaridade (anos)	8 ± 4	9 ± 4	0,016
Brancos (n, %)	59 (86,8%)	127 (78,4%)	0,308
Renda familiar < 5 salários (n, %)	63 (91,3%)	123 (72,8%)	0,006
SUS (n, %)	61 (89,7%)	123 (75,5%)	0,014
Fatores de risco			
HAS (n, %)	44 (62,0%)	88 (50,6%)	0,114
DM (n, %)	20 (28,2%)	35 (20,1%)	0,170
TABAG (n, %)	46 (64,8%)	77 (44,4%)	0,005
DSLP (n, %)	25 (35,2%)	41 (23,6%)	0,062
HF+ (n, %)	19 (26,8%)	45 (25,9%)	0,885
**História clínica pregressa**			
IAM prévio (n, %)	16 (22,9%)	36 (21,3%)	0,791
ICP prévio (n, %)	12 (17,1%)	28 (16,6%)	0,914
Angina (n, %)	21 (30,0%)	53 (31,4%)	0,836
ICC (n, %)	5 (7,1%)	7 (4,2%)	0,350
Depressão (n, %)	26 (37,7%)	19 (11,3%)	< 0,001
DPOC (n, %)	5 (7,1%)	3 (1,8%)	0,037
IRC (n, %)	1 (1,4%)	2 (1,2%)	0,877
AVC prévio (n, %)	4 (5,7%)	10 (5,9%)	0,952
**Evolução clínica**			
Arritmia (n, %)	4 (5,8%)	3 (1,8%)	0,105
IAM recorrente (n, %)	1 (1,4%)	1 (0,6%)	0,526
AVC (n, %)	0	1 (0,6%)	–
Óbito intra-hospitalar (n, %)	3 (4,3%)	1 (0,6%)	0,045

* Qui-quadrado ou t de student para amostras independentes.

IMC: índice de massa corporal; SUS: Sistema Único de Saúde; HAS: hipertensão arterial sistêmica; DM: diabete melito; TABAG: tabagismo; DSLP: dislipidemia; HF+: história familiar positiva para doença arterial coronariana; IAM: infarto agudo do miocárdio; ICP: intervenção coronariana percutânea; AVC: acidente vascular cerebral; ICC: insuficiência cardíaca congestiva; DPOC: doença pulmonar obstrutiva crônica; IRC: insuficiência renal crônica.

**Figura 2:**
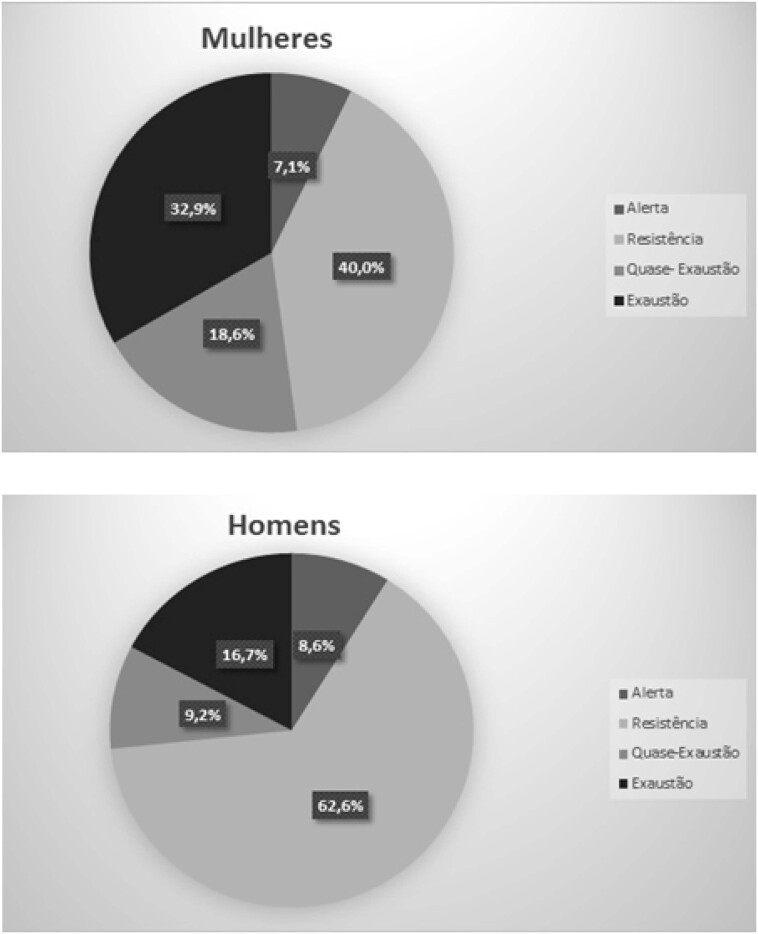
Comparação das fases do estresse entre mulheres e homens.

Na avaliação das fases do estresse, as mulheres se mostraram mais na de quase exaustão (18,6% *versus* 9,2%; p = 0,041) e exaustão (32,9% *versus* 16,7%; p = 0,005), e os homens, mais na de resistência (40,0% versus 62,6%; p < 0,001). Houve prevalência semelhante na fase de alerta (7,1% *versus* 8,6%; p = 0,703) e o predomínio de sintomas físicos (77,1% *versus* 73,6%; p = 0,586) em ambos os grupos. A Figura 2 mostra a distribuição desses percentuais.

## Discussão

Nesse estudo, observou-se que a prevalência de estresse entre pacientes com IAM foi de 74%, semelhante às descritas para pacientes cardiopatas, que variam entre 72 e 85% em hipertensos.^14^^,^^19^ Há, portanto, alta prevalência de estresse entre os pacientes acometidos de infarto e submetidos à intervenção coronariana percutânea primária.

As mulheres apresentaram mais estresse do que os homens, conforme já descrito na literatura. No estudo de Calais e Lipp,^12^ por exemplo, sobre as diferenças de sexo e escolaridade na manifestação da doença, as autoras, utilizando o ISSL, encontraram uma prevalência de 79,30% nas mulheres, enquanto o percentual de homens com sintomas de estresse era 51,72%. Já em uma pesquisa com magistrados da justiça do trabalho,^13^ também utilizando o mesmo instrumento, 82% das juízas e 56% dos juízes estavam estressados, o que mostra uma diferença significativa entre os sexos. Embora essas pesquisas descrevam percentuais superiores de estresse nas mulheres, nenhuma delas avaliou se o sexo feminino era um preditor independente, sendo esse um diferencial deste estudo. Nele, foi encontrada uma prevalência maior em ambos sexos, 89% em mulheres e 70% em homens. Assim, a análise multivariada demonstrou que o sexo feminino tem as chances de estresse quase triplicadas.

O tipo de assistência à saúde também foi um preditor independente para a ocorrência da doença, sendo os pacientes atendidos pelo SUS os mais acometidos. Dados semelhantes foram encontrados no estudo de Santos et al.,^20^ no qual mais pacientes do grupo SUS apresentaram estresse em relação ao grupo dos conveniados, o que sugere a maior vulnerabilidade dos pacientes da assistência pública, provavelmente devido a menor *status* socioeconômico e menos escolaridade e renda, fatores associados a maiores níveis de estresse.^21^ Neste estudo, contudo, não foram realizadas subanálises para verificar as possíveis diferenças entre o grupo SUS e convênios, sendo esta uma possibilidade para pesquisas futuras.

Na análise somente com os pacientes estressados conforme os sexos, observou-se que as mulheres com estresse apresentam mais história prévia de depressão. Além disso, tem sido descrito que elas são significativamente mais propensas a terem diagnóstico prévio dessa doença^22^^,^^23^ em relação aos homens, principalmente no climatério,^24^ e há um crescente interesse em saber se a maior diferença entre os sexos na taxa de depressão pode ser o aumento da exposição e reatividade aos estressores.^25^ Tem sido observado também que as mulheres são substancialmente mais propensas a serem vítimas de experiências traumáticas, como abuso sexual e agressão, do que os homens, e que tais ocorrências, especialmente na infância ou adolescência, são comumente preditivas de episódios depressivos.^25^

Condições estressantes crônicas, como pobreza e pais solteiros (monoparentalidade), excedem nas mulheres em comparação aos homens, e essas circunstâncias são frequentemente associadas à depressão.^25^ Experimentar doenças crônicas e atuar como cuidadores de cuidados primários para parentes doentes também parece ser uma experiência vivenciada mais por mulheres do que homens, o que também está ligado à doença.^25^^,^^26^ É descrito ainda que “tensões crônicas” relacionadas a papéis femininos na forma de redução de poder e tomada de decisão, como falta de afirmação em relacionamentos íntimos, sobrecarga de papéis, desigualdades domésticas e cuidados com crianças, foram preditores de depressão ao longo do tempo e mediaram parcialmente as diferenças de gênero na depressão.^25^^,^^27^

No estudo de Hammen et al.,^28^ que avaliou a relação entre estresse e depressão nas mulheres, o aparecimento de depressão foi significativamente associado ao estresse crônico e agudo. Havia também uma tendência, consistente com um efeito sensibilizador, de que o estresse crônico moderasse os efeitos de eventos estressores agudos na depressão maior, de modo que altos níveis da forma crônica amplificaram o impacto de eventos agudos. Em contraste, a associação entre estresse agudo e depressão foi menor entre mulheres com níveis mais baixos do crônico. Os resultados confirmam a importância de levar em conta os efeitos do estresse crônico na relação entre estresse e depressão em mulheres.

Ainda na análise somente com os pacientes estressados conforme os sexos, observou-se que as mulheres estressadas são mais tabagistas que os homens e com mais história de DPOC. No entanto, dados epidemiológicos mostraram que há maior prevalência de tabagismo no sexo masculino,^29^ e a quantidade de doenças relacionadas ao tabaco superou o dobro nos homens quando comparados às mulheres, com concentração em DPOC, IAM, pneumonia e AVC.^30^ Desse modo, a maior ocorrência de tabagismo e DPOC nas mulheres, quando considerada somente a população estressada, chama a atenção, sendo o estresse um fator que pode estar influenciando esses achados.

Segundo Bussoletto,^15^ ao lidar com os desafios do dia a dia e na busca por relaxamento e recompensa, os pacientes recorrem ao tabagismo e à alimentação inadequada como estratégias, o que piora não apenas o nível de estresse, mas também a doença cardíaca. Essa premissa pode ser verdadeira porque, embora não haja significância estatística, o percentual de dislipidemia foi maior também nas mulheres. Assim, considera-se que, embora as mulheres não tivessem mais idade em relação aos homens na amostra (fator conhecidamente associado à mortalidade em mulheres),^31^ esse acúmulo de fatores de risco, destacando ainda menores escolaridade e renda, pode ter contribuído para o maior percentual de óbitos intra-hospitalares encontrado no sexo feminino. Contudo, salienta-se que esse estudo não foi desenhado para avaliar a mortalidade.

Embora as mulheres sofram mais com o estresse emocional, a maioria dos estudos, principalmente com pacientes isquêmicos, incluíram poucas delas. Assim, Lucinda et al.,^14^ analisando o estresse em pacientes após IAM e ativos no mercado de trabalho, descreveram que 71% da amostra encontravam-se na fase de resistência (91% da amostra eram compostos por homens, e apenas 9%, por mulheres). De maneira idêntica, Bussoletto^15^ encontrou 78,6% da sua amostra na fase de resistência, e seu estudo incluiu 83,87% de homens e 16,13% de mulheres. Em nosso estudo, foram encontrados 57,6% dos participantes na fase de resistência, com 76% de homens, ou seja, um percentual maior que o de mulheres (24%). Assim, foi possível observar as diferenças nas fases do estresse entre homens e mulheres; estas se apresentaram predominantemente nas fases de quase exaustão e exaustão, ou seja, de estresse crônico. Esses resultados corroboram o estudo de Wottrich et al.,^19^ que, utilizando também o ISSL, analisou o estresse em pacientes hipertensos conforme o gênero. A autora também observou que a maioria das mulheres estava na fase de exaustão (41,4% *versus* 15,2%), enquanto os homens estavam na fase de resistência (60,6%). Curiosamente, essa amostra de 103 pacientes foi composta, predominantemente (70%), por mulheres.

### Limitações

As mulheres do nosso estudo apresentaram menores escolaridade e renda familiar do que os homens. Assim, supõem-se que as condições socioeconômicas também estejam mediando a presença de estresse, além de contribuir para as doenças cardiovasculares. Entretanto, não se sabe se essas mulheres apresentaram menor renda por serem sós, viúvas e sem companheiros. Também não são conhecidos outros fatores psicossociais, como número e idade dos filhos e netos, nem condições de vulnerabilidade social. Não há informações sobre a rede de apoio familiar, o que significa que podem existir outros aspectos relacionados aos achados.

Este estudo utilizou, para a avaliação do estresse, o ISSL, que mensura a presença de sintomas nas últimas 24 horas, na última semana e no último mês, podendo existir viés de informação por parte dos entrevistados ao responderem o questionário.

## Conclusão

O estudo evidenciou que ser do sexo feminino e utilizar o SUS são preditores independentes de risco para o estresse em pacientes com infarto recente. As mulheres encontravam-se na terceira e quarta fases da doença, ou seja, em situações de estresse duradouras. Elas também apresentaram menores escolaridade e renda familiar, além de parecerem minimizar o estresse cotidiano com o cigarro. O sexo feminino ainda mostrou mais depressão do que o masculino. Esses resultados podem auxiliar no desenvolvimento de estratégias específicas de gênero para a prevenção e a promoção da saúde, visando minimizar os efeitos do estresse nos pacientes.
