# Supplementary material for: Um Olhar sobre o Stress nas Mulheres com Infarto Agudo do Miocárdio
Source: Arq Bras Cardiol. 2020 Oct 13;115(4):649–57. [Article in Portuguese] doi: 10.36660/abc.20190282 (PMC8386975; doi:10.36660/abc.20190282)
Supplement: Translation

# Translation: Stress in Women with Acute Myocardial Infarction: A Closer Look

**Authors:** Karine Schmidt, Aline da Silva Lima, Kelly Rocha Schmitt, Maria Antonieta Moraes, Marcia Moura Schmidt

**Affiliations:** 1 Porto AlegreRS Brazil Instituto de Cardiologia, Porto Alegre, RS – Brazil

**Keywords:** Women, Myocardial Infarction, Stress, Psychological, Coronary Artery Disease, Vascular Diseases, Risk Factors

## Abstract

**Background:**

Women seem to be more susceptible to psychosocial stress than men, and stress is associated with worse outcomes after acute myocardial infarction (AMI).

**Objectives:**

To investigate whether the female gender is an independent predictor of risk for stress and to compare stress levels between women and men after AMI.

**Methods:**

Cross-sectional study of a case series. Patients aged 18 to 65 years who were treated for AMI at the study facility between January 2017 and June 2018 were eligible. The presence of stress was assessed using Lipp's Stress Symptoms Inventory for Adults (ISSL), which categorizes stress into four phases (alertness, resistance, near-exhaustion, and exhaustion), through a list of physical and psychological symptoms. Data were analyzed using SPSS Version 24.0. The significance level was set at p<0.05.

**Results:**

Of the 330 respondents, 89% of women and 70% of men experienced stress. The female gender was associated with nearly threefold higher odds of experiencing stress (EXP (B)2.79, p = 0.02). Regarding the phases of stress, women were more often in the near-exhaustion and exhaustion phases, while men were more often in the resistance phase.

**Conclusions:**

This study showed that women are most often in the third and fourth phases of stress, i.e., in situations of long-standing psychosocial stress. These findings can assist in the development of gender-specific strategies for health promotion and disease prevention, aiming to minimize the effects of stress in this population.

## Introduction

Selye was the first to identify stress as a set of reactions that the body exhibits when it is required to adapt to a situation by exerting an effort.^1^ Stress is thus understood as a reaction to any stressful event, or *stressor*, and can trigger behavioral, psychological, and physical symptoms.^2^

In the intercontinental INTERHEART study,^3^ conducted with 11,119 cases and 13,648 controls across 52 countries, the presence of stressors was shown to double the risk of acute myocardial infarction (AMI). Data from a U.S. National Health Interview Survey^4^ confirm these findings, and demonstrate that stress and psychological distress can double the risk of AMI (OR: 2.0; 95%CI, 1.4 to 3.0).

Chronic psychological stress produces the overactivation of the sympathetic nervous system, which exacerbates coronary atherosclerosis and endothelial dysfunction.^5^^,^^6^ In the long term, this may increase the risk of coronary events and death.^7^

Studies have shown that women are more susceptible to psychosocial stress.^8^^,^ ^9^ Women's daily lives involve multiple social and family roles, which makes them as much as or more susceptible to cardiovascular diseases than men.^10^ Acute myocardial infarction (AMI) and stroke are the leading causes of death in women over the age of 50. In this age group, there are more cardiovascular deaths than by any other cause, including breast cancer.^11^

In Brazilian studies, younger women have been found to experience a higher frequency of stress symptoms compared to men.^12^^,^^13^ However, in studies on stress and ischemic heart disease, women are less prevalent and older than males.^14^^,^^15^ Within this context, the objective of the present study was to investigate predictors of stress, mainly to investigate whether female gender is an independent predictor of stress risk, as well as to compare sociodemographic and clinical characteristics, medical history, in-hospital events and stress levels between women and men with acute myocardial infarction.

## Methods

### Design and Participants

Cross-sectional study of a case series conducted over an 18-month period. Patients were included according to the following criteria: Age 18 to 65 years (i.e., working age); treated for acute myocardial infarction with ST-segment elevation (STEMI), within 12 hours of symptom onset, at a cardiology referral center. According to the 5^th^ Brazilian Society of Cardiology Guideline on the Treatment of STEMI (2015),^16^ AMI is defined as an acute ischemic syndrome with ST-segment elevation >1.0 mm on contiguous ECG leads. Increased and/or decreased levels of cardiac markers, especially troponin (>99^th^ percentile), are essential for the diagnosis. Exclusion criteria: Prolonged time from symptom onset until hospital arrival; need for mechanical ventilation; development of delirium; previous history of dementia, cognitive impairment; or known psychiatric conditions, according to the attending physician, which would preclude comprehension of the informed consent form.

Participants were interviewed during the first 48 hours of hospitalization. Data on sociodemographic variables, past medical history, and risk factors for ischemic heart disease were collected. Race was self-reported by the participants. Hypertension was defined as a previous diagnosis or current use of antihypertensive agents. Dyslipidemia was considered present in patients with a previous diagnosis or those currently using lipid-lowering drugs. Diabetes mellitus was defined by a documented fasting glucose >126 mg/dL on two occasions or a history of use of insulin or antidiabetic agents. Family history of CAD was considered positive if any first-degree relatives had a history of AMI or sudden cardiovascular death before age 55 (men) or 65 (women). BMI (body mass index) was calculated using the self-reported weight and height data. Depression was defined by the occurrence of at least one major depressive episode requiring pharmacological treatment. Angina was defined as pain or discomfort in the anterior chest, epigastric region, jaw, shoulder, back or upper limbs, triggered or worsened by physical activity or emotional stress (class II or higher), according to the Stable Coronary Disease Guidelines.^17^ Medical records were checked for any intercurrent events occurring during hospitalization.

#### Instruments

The presence of stress was assessed using the LIPP's Stress Symptoms Inventory for Adults (ISSL),^2^ which has been validated by the Brazilian Federal Board of Psychology. The ISSL consists of a list of 53 physical and psychological symptoms, divided into time periods: in the last 24 hours, in the last week, and in the last month. In addition to detecting the presence or absence of stress, the instrument categorizes stress into four phases: alertness (corresponding to the score obtained for symptoms present in the last 24 hours), resistance, near-exhaustion (corresponding to the scores for the last week), and exhaustion (corresponding to symptoms that were present in the month preceding the interview). This instrument thus allows the diagnosis of stress, establishing the stage of stress the respondent is currently experiencing, and whether there is a predominance of physical, psychological, or mixed symptoms.

#### Phases of Stress

The alertness phase is characterized by sympathetic nervous system reactions, elicited by the initial perception of the stressor. The resistance phase develops when a stressor persists over time – causing the person to resist and find the strength to continue coping with the stress, although the symptoms remain present. In the near-exhaustion phase, the actual pathological process begins, and organs that have a greater genetic or acquired vulnerability start to show signs of deterioration. If the stress is not relieved, whether by removal of the stressor or the use of coping strategies, the exhaustion phase is reached. Various conditions arise, such as stress ulcers, gingivitis, psoriasis, hypertension, depression, and anxiety, among others.^2^

#### Ethical Considerations

This study was approved the institutional Research Ethics Committee (CAAE: 62727416.5.0000.5333). All participants agreed to participate in the study and signed the Free and Informed Consent Form (ICF), pursuant to the provisions of Brazilian National Health Council Resolutions 466/12 and 510/2016.

#### Sample Size

The sample size was calculated using the WinPepi software, version 11.29. Considering that the difference between the proportion of men and women with stress ranges from 30%^12^ to 50%^13^ and that 70% of patients with acute myocardial infarction are males,^18^ for a significance level of 0.05 and a statistical power of 80%, the minimum sample size would comprise 194 participants (114 men, 80 women).

#### Statistical Analysis

Data were entered into a Microsoft Excel database and analyzed using the Statistical Package for Social Sciences (SPSS), Version 24.0. The Kolmogorov–Smirnov test was used to verify the normality of the variables. Continuous variables were expressed as mean and standard deviation, and categorical variables, as absolute and relative frequencies. The multivariate logistic regression was performed for the predictors of stress. Variables that reached p<0.10 in the bivariate analysis were retained for the multivariate model. A *t*-test for independent samples or chi-square test was used for comparison of variables between participants with and without stress and by gender. A p-value < 0.05 was considered statistically significant. The prevalence of stress, its phases, and symptoms were presented as relative frequencies and compared with the chi-square test as necessary, following the rules of the ISSL Instrument, in which the responses are categorized.^2^

## Results

Patients were included consecutively from August 2017 through June 2018. According to the study flowchart diagram (Figure 1), of the 632 patients with AMI assessed for eligibility, 211 did not meet the inclusion criteria, 32 were excluded according to the established criteria, 40 died, and 19 were excluded because they were not interviewed within the first 48 hours of hospitalization. Thus, the final sample consisted of 330 participants.

**Figure 1:** Study flowchart diagram. January 2017 to June 2018.

Of these 330 respondents, 80 were women and 250 were men. Overall, 74% were experiencing stress (89% of women and 70% of men). Table 1 shows the clinical characteristics of the sample, stratified by the presence or absence of stress. We observed that those respondents with stress were mostly women, with fewer years of formal education, and had been admitted under the care of the Unified Health System (SUS). There were no significant differences between the risk factors; however, regarding the past medical history, patients with stress were more likely to have angina and depression. There were no differences regarding complications and intercurrent events occurring during hospitalization in these patients.

**Table 1:** Difference in stress prevalence between genders

Characteristics	Overall n=330	Stress n=245	No stress n=85	p*
Sociodemographic characteristics				
Female gender, n%	80 (24.2%)	71 (29.0%)	9 (10.6%)	0.001
Age, years	54.6 ± 7.7	54.4 ± 8.10	55.4 ± 7.0	0.28
BMI, kg/m²	27.8 ± 5.1	27.6 ± 5.0	28.2 ± 5.5	0.33
Level of schooling, years	9.0 ± 4.0	8.7 ± 4.4	9.9 ± 3.3	0.02
Caucasian, n (%)	249 (81.0%)	186 (81.0%)	63 (80.8%)	0.32
Household income <5× minimum wage, n (%)	243 (76.0%)	186 (78.2%)	57 (69.5%)	0.11
Unified Health System, n (%)	231 (76.0%)	184 (79.7%)	47 (64.4%)	0.008
**Risk factors**				
HTN, n (%)	180 (54.5%)	132 (54.0%)	48 (56.2%)	0.68
DM, n (%)	75 (22.7%)	55 (22.4%)	20 (23.5%)	0.84
Smoking, n (%)	120 (49.0%)	120 (49.0%)	38 (44.7%)	0.09
Dyslipidemia, n (%)	68 (27.8%)	68 (27.8%)	21 (24.7%)	0.58
FH+, n (%)	81 (24.8%)	62 (25.6%)	19 (22.4%)	0.55
**Past medical history**				
Prior AMI, n (%)	64 (19.7%)	52 (21.7%)	12 (14.1%)	0.13
Prior PCI, n (%)	48 (14.8%)	40 (16.7%)	8 (9.4%)	0.10
Prior stroke, n (%)	20 (6.2%)	14 (5.8%)	6 (7.1%)	0.68
CHF, n (%)	16 (5.0%)	12 (5.1%)	4 (4.7%)	0.90
Angina, n (%)	87 (26.8%)	74 (30.8%)	13 (15.3%)	0.005
COPD, n (%)	9 (2.8%)	9 (3.8%)	0	−
CKD, n (%)	4 (1.2%)	3 (1.3%)	1 (1.2%)	0.95
Depression, n (%)	51 (15.8%)	45 (19.0%)	6 (7.1%)	0.01
**In-hospital events**				
Arrhythmia, n (%)	9 (2.8%)	7 (3.0%)	2 (2.4%)	0.75
Recurrent AMI, n (%)	2 (0.6%)	2 (0.9%)	0	–
Stroke, n (%)	1 (0.3%)	1 (2.5%)	0	–
Death, n (%)	4 (1.3%)	4 (1.7%)	0	–

AMI: acute myocardial infarction; BMI: body mass index; CHF: congestive heart failure; CKD: chronic kidney disease; COPD: chronic obstructive pulmonary disease; DM: diabetes mellitus; FH+: family history of coronary artery disease; HTN: hypertension; PCI: percutaneous coronary intervention.

* Chi-square or Student's t-test for independent samples.

After the bivariate analysis of the characteristics of patients with *vs*. without stress, we selected those with p<0.10 for inclusion in the multivariate analysis model (Table 2). The results showed that female gender and being admitted under the Brazilian public healthcare system (Unified Health System) were independent predictors of stress. The relative risk of exposure to stress—Exp(B)—in women was 2.79 (95% CI 1.21 to 6.40, p=0.02), which suggests that being female nearly triples one's odds of experiencing stress.

**Table 2:** Multivariate analysis of independent predictors of stress

Characteristics	Exp(B)	95% confidence interval	p
Females	2.79	1.21-6.40	0.02
Level of schooling	0.98	0.91-1.06	0.66
Angina	1.90	0.94-3.87	0.07
Depression	1.52	0.57-4.02	0.40
Smoking	1.06	0.60-1.86	0.84
Unified Health System	1.93	1.00-3.70	0.05

The analysis by gender included only those patients with stress, i.e., a total of 245 participants (71 women and 174 men). As seen in Table 3, women with stress had a lower level of schooling than men, and a greater proportion of them had a lower household income. Women were also hospitalized under the care of the public system more frequently than men (89.7% vs 75.5%, p=0.014). There were no significant differences in relation to age or body mass index (BMI). Regarding the risk factors, smoking was more prevalent among women than men. Hypertension, diabetes, and family history of coronary heart disease were similar in both groups. There was an apparent trend toward dyslipidemia among women. Women were also more likely than men to have a history of depression and chronic obstructive pulmonary disease (COPD). Regarding the clinical course, the in-hospital mortality was higher in women.

**Table 3:** Comparison of profiles between women and men with stress

Characteristics	Women n=71 (89%)	Men n=174 (70%)	p*
**Sociodemographic characteristics**			
Age, years	55 ± 9	54 ± 7	0.667
BMI, kg/m²	27 ± 7	28 ± 4	0.168
Level of schooling, years	8 ± 4	9 ± 4	0.016
White ethnicity, n (%)	59 (86.8%)	127 (78.4%)	0.308
Household income <5× minimum wage, n (%)	63 (91.3%)	123 (72.8%)	0.006
Unified Health System, n (%)	61 (89.7%)	123 (75.5%)	0.014
**Risk factors**			
HTN, n (%)	44 (62.0%)	88 (50.6%)	0.114
DM, n (%)	20 (28.2%)	35 (20.1%)	0.170
Smoking, n (%)	46 (64.8%)	77 (44.4%)	0.005
Dyslipidemia, n (%)	25 (35.2%)	41 (23.6%)	0.062
FH+, n (%)	19 (26.8%)	45 (25.9%)	0.885
**Past medical history**			
Prior AMI, n (%)	16 (22.9%)	36 (21.3%)	0.791
Prior PCI, n (%)	12 (17.1%)	28 (16.6%)	0.914
Angina, n (%)	21 (30.0%)	53 (31.4%)	0.836
CHF, n (%)	5 (7.1%)	7 (4.2%)	0.350
Depression, n (%)	26 (37.7%)	19 (11.3%)	<0.001
COPD, n (%)	5 (7.1%)	3 (1.8%)	0.037
CKD, n (%)	1 (1.4%)	2 (1.2%)	0.877
Prior stroke, n (%)	4 (5.7%)	10 (5.9%)	0.952
**Intercurrent events**			
Arrhythmia, n (%)	4 (5.8%)	3 (1.8%)	0.105
Recurrent AMI, n (%)	1 (1.4%)	1 (0.6%)	0.526
Stroke, n (%)	0	1 (0.6%)	−
In-hospital death, n (%)	3 (4.3%)	1 (0.6%)	0.045

AMI: acute myocardial infarction; BMI: body mass index; CHF: congestive heart failure; CKD: chronic kidney disease; COPD: chronic obstructive pulmonary disease; DM: diabetes mellitus; FH+: family history of coronary artery disease; HTN: hypertension; PCI: percutaneous coronary intervention.

* Chi-square or Student's t-test for independent samples.

Assessment of the phases of stress showed that women were more likely than men to be in the near-exhaustion (18.6% vs. 9.2%, p= 0.041) and exhaustion (32.9% vs. 16.7%, p = 0.005) phases, while men were more likely to be in the resistance phase (40.0% vs. 62.6%, p<0.001). The prevalence of the alertness phase (7.1% vs. 8.6%, p=0.703) and a predominance of physical symptoms (77.1% vs. 73.6%, p=0.586) were similar in the two groups. Figure 2 illustrates the distribution of these percentages.

**Figure 2:** Comparison of stress phases in women and men.

## Discussion

In this study, the prevalence of stress in patients admitted with acute myocardial infarction was 74%. The overall prevalence of stress was similar to that described in previous studies of patients with cardiovascular disease, ranging from 72%^14^ up to 85% in patients with hypertension.^19^ Therefore, there is a high prevalence of stress in patients undergoing primary percutaneous coronary intervention for the treatment of myocardial infarction.

Women experienced more stress than men, as described elsewhere in the literature. In a study by Calais and Lipp^12^ on the differences in stress manifestations by gender and level of schooling, the authors, using the ISSL, found an overall stress prevalence of 79.30% in women and 51.72% in men. In a survey of magistrate court employees^13^ using the same instrument, 82% of female and 56% of male magistrate employees were stressed, demonstrating a significant difference between the genders. Although these surveys described higher percentages of stress in women, neither of them evaluated whether female gender alone was an independent predictor, which is a strength of our study. We found a higher prevalence in both genders, although it was higher in women (89% and 70%, respectively). The multivariate analysis showed that female gender was associated with nearly threefold higher odds of stress.

The healthcare model was also an independent predictor for the occurrence of stress, with patients treated by the Unified Health System (SUS) being the most affected. Similar data were found in a study by Santos et al.^20^ in which more patients in the SUS group showed more stress as compared to health-insured patients. This suggests that patients treated by the public system are more vulnerable, probably due to the lower socioeconomic status of these patients, lower level of schooling, and lower income—all factors associated with higher levels of stress.^21^ In our study, however, we did not perform any subgroup analyses to check for possible differences between the SUS group and patients with private health insurance. This remains a possibility for future research.

On a subgroup analysis restricted to patients with stress, stratified by gender, we found that women with stress were more likely to have a history of depression. It has been described that women are significantly more likely to have a history of depression diagnosis,^22^^,^^23^ especially during menopause,^24^ as compared to men. There is a growing interest in finding out whether this gender difference in rates of depression might be driven by greater exposure and reactivity to stressors.^25^ Women are substantially more likely to be victimized by traumatic experiences (such as sexual abuse and assault), than men. Such experiences, especially in childhood or adolescence, are commonly predictive of depressive episodes.^25^ Chronic stressors often associated with depression, such as poverty and single parenthood, are far more common in women than in men.^25^ Experiencing chronic illness and being the primary caregiver for sick family members also seem to be experiences far more common for women than for men. These conditions are likewise associated with depression.^25^^,^^26^ Studies have suggested that “chronic strain” related to traditional female roles, manifested by reduced agency and decision-making (such as submissiveness in intimate relationships, role overload, domestic inequalities, childcare responsibilities), were predictive of depression over time and partially mediated gender differences in depression.^25^^,^^27^

In a study by Hammen et al.^28^ evaluating the stress-depression relationship in women, the onset of depression was significantly associated with chronic and acute stress. There was a trend, consistent with a sensitizing effect, that chronic stress would moderate the effects of acute stressful events in major depression, so that high levels of chronic stress amplified the impact of acute events; conversely, the association between acute stress and depression was lower among women with lower levels of chronic stress. These findings confirm the importance of taking into account the effects of chronic stress on the stress-depression relationship in women.

Our subgroup analysis restricted to stressed patients, and stratified by gender, found that stressed women smoke more and are more likely to have a history of COPD than men. Epidemiological data support a higher prevalence of smoking in men,^29^ and rates of tobacco-related illnesses in men are more than double those in women, with particular focus on COPD, AMI, pneumonia, and stroke.^30^ The higher prevalence of smoking and COPD in women when we consider only the stressed population warrants further analysis. Stress itself may be influencing these findings.

According to Bussoleto,^15^ when dealing with day-to-day challenges and seeking relaxation and rewards, patients resort to smoking and inadequate nutritional strategies. This not only worsens their level of stress, but also worsens heart disease. This premise may have been held true in our study, because, although it was not powered to detect significance, the percentage of dyslipidemia was higher in women than in men. We consider that, although women were not older than men in this sample (a factor known to be associated with mortality in women),^31^ this buildup of risk factors—which also highlighted the lower level of schooling and income of women as compared to men – may have contributed to the higher percentage of in-hospital deaths found in women. Nevertheless, we must emphasize that this study was not designed to assess mortality.

Although women experience more emotional stress, most studies, especially those of ischemic patients, have included few women. Lucinda,^14^ analyzing stress in post-AMI patients who remained active in the job market, found that 71% of the sample was in the resistance phase, with 91% of the sample consisting of men and only 9% of women. Likewise, Bussoleto^15^ found 78.6% of his sample in the resistance phase, in a study that included 83.87% men and 16.13% women. In our study, 57.6% of participants were in the resistance phase, including 76% of men, that is, a higher percentage than among women (24%). Thus, we were able to observe differences in the phases of stress between men and women. Women were predominantly in the near-exhaustion and exhaustion phases, i.e., in the phases of chronic stress. These results corroborate the findings of Wottrich,^19^ who also used the ISSL to analyze stress in hypertensive patients according to gender. The author also found that women were mostly in the exhaustion phase of stress (41.4% vs. 15.2%), while men were in the resistance phase (60.6%). Interestingly, this sample of 103 patients consisted predominantly (70%) of women.

### Limitations

The women in the present study had lower level of schooling and lower household income than the men. Thus, it is assumed that socioeconomic conditions also act as mediators in the presence of stress, in addition to contributing to cardiovascular diseases. However, our design precludes any inference as to whether women had lower income because they lived alone or were widowed. We are also unaware of any other psychosocial factors, such as the number and age of children and grandchildren, or about social vulnerability conditions. We have no information about their family support networks. In other words, there may be other factors related to our findings.

This study used Lipp's Stress Symptoms Inventory for Adults (ISSL), an instrument that measures the presence of symptoms in the last 24 hours, in the last week, and in the last month. However, it is subject to reporting bias by the respondents when completing the questionnaire.

## Conclusion

In this study, female gender and being admitted to the hospital under the Unified Health System (SUS) were independent predictors of risk for stress in patients with recent AMI. Women were most often in the third and fourth phases of stress, i.e., in situations of long-standing psychosocial stress. Women also had fewer years of schooling and lower household income and appeared to use tobacco smoking quite often as a coping strategy. Depression was also more prevalent in women than in men. These findings can assist in the development of gender-specific strategies for health promotion and disease prevention, aimed at minimizing the effects of stress on patients.
